# Urétéro-hydronéphrose avec rupture du fornix sur syndrome de jonction pyélo-urétérale

**DOI:** 10.11604/pamj.2024.48.17.42481

**Published:** 2024-05-21

**Authors:** Idriss Ziani, Ahmed Ibrahimi

**Affiliations:** 1 Service de Chirurgie Urologique A, CHU de Rabat, Rabat, Maroc,; 2 Faculté de Médecine et de Pharmacie, Université Mohammed V de Rabat, Rabat, Maroc

**Keywords:** Fornix rénal, rupture, syndrome de jonction, Renal fornix, rupture, junction syndrome

## Abstract

Rupture of the pyelocalyceal cavities also known as Renal Fornix Rupture (RFR) with urine extravasates into the retroperitoneum is a rare urological complication, most frequently associated with acute urinary tract obstruction induced by a calculus. We here describe an exceptional aetiology of forniceal rupture, i.e. pyeloureteral junction. To our knowledge, this is the first observation reported in the literature of such a complication. Common etiologies responsible for RFR are posterior urethral valves in the neonatal period, prostatic hyperplasia, or traumatic causes, mainly lithiasis. We here report the case of a young 19-year-old patient presenting with febrile low back pain. Uroscanner showed a rupture of the excretory system with extravasation of the contrast medium and large urinoma (A). Clinical and biological outcomes were good with complete regression of the urinoma on a follow-up CT scan (B). Following DJ stent removal, Diethylene-Triamine-Pentaacetate (DTPA) scintigraphy objectified pyelo-ureteral junction syndrome. The patient underwent laparoscopic surgery with favorable outcome (C, D).

## Image en médecine

La rupture des cavités pyélo-calicielles encore dénommée rupture du fornix rénal (RFR) avec extravasation rétropéritonéale d'urine est une complication urologique rare le plus fréquemment en lien avec une obstruction aiguë des voies urinaires par un calcul. Nous décrivons par notre observation une étiologie exceptionnelle d'une rupture du fornix à savoir la jonction pyélo-urétérale. À notre connaissance, c'est la première observation rapportant une telle complication dans la littérature. Les étiologies habituelles responsables de RFR sont les valves de l'urètre postérieur en période néonatale, les hyperplasies prostatiques ou les causes traumatiques et principalement la pathologie lithiasique. Nous rapportons le cas d'un jeune patient de 19 ans consulté pour lombalgie fébrile. L'uro-scanner objective une rupture des voies excrétrices avec extravasation du produit de contraste et un urinome de grande abondance (A). Le patient a bénéficié en urgence d'un drainage par sonde double J avec une antibiothérapie probabiliste par ceftriaxon et gentamycine. Une bonne évolution clinico-biologique avec une régression complète de l'urinome sur le scanner de contrôle (B). Suite au retrait de la sonde double J dans le cadre du bilan étiologique, une scintigraphie au DTPA a objectivé un syndrome de la jonction pléyo-urétérale. Le patient a bénéficié par la suite d'une cure du syndrome de jonction par voie laparoscopique avec évolution favorable (C, D).

**Figure 1:**
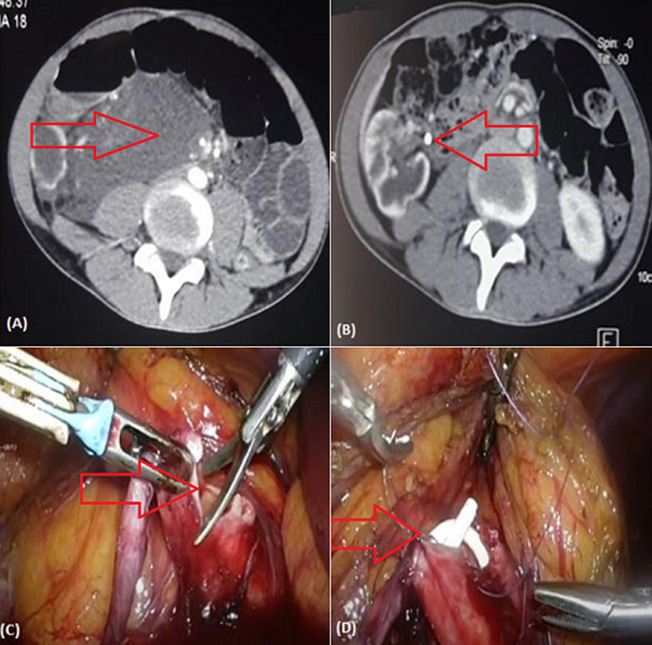
scanner de la rupture du fournix avec urinome (A); scanner objectivant une nette régression de l'urinome après drainage par sonde double J (B); image per-opératoire objectivant la cure laparoscopique du syndrome de jonction pyélo-urétrale (C, D)

